# Effect of adhesive strategy of universal adhesives in noncarious cervical lesions – an updated systematic review and meta-analysis

**DOI:** 10.1038/s41405-022-00124-6

**Published:** 2023-02-13

**Authors:** Krisha Doshi, M. S. Nivedhitha, Pradeep Solete, Delphine Pricilla Antony S, Arthi Balasubramaniam, Benoy Jacob, Riluwan Siddique

**Affiliations:** 1 Department of Conservative Dentistry and Endodontics, Saveetha Dental College and Hospitals, Saveetha Institute of Medical And Technical Sciences (SIMATS), Chennai, Tamil Nadu India; 2 Department of Public Health Dentistry, Saveetha Dental College, Saveetha University, Chennai, India

**Keywords:** Bonded restorations, Pulp conservation

## Abstract

**Objective:**

To determine the effect of adhesive strategy (total etch or self-etch) of universal adhesives in non-carious cervical lesions.

**Data source:**

A search was made in PubMed, Scopus, Cochrane, Web Of Science, Open Gray, Clinical Registries.

**Data selection:**

Randomized Controlled Clinical Trials, studies on non-carious cervical lesions restored using Universal Adhesives, and studies in which universal adhesives have been used in total etch and self -etch strategies were included in this systematic review.

**Data extraction:**

A total of 17 articles were included in the systematic review and 13 in the meta-analysis. Meta-analysis was conducted to assess the clinical performance of NCCLs in terms of retention, marginal adaptation, marginal discoloration, secondary caries and post-operative sensitivity at 18, 24, 36 month follow-up using USPHS as well as FDI criteria, separately.

**Data synthesis:**

Overall there was no significant difference between total etch and self etch adhesive strategies for any of the five outcome measures using either the FDI or the USPHS criteria. p > 0.05, 95% CI, I^2^ value of 0%. A strongly suspected publication bias in the retention domain was seen at 18 month follow up under FDI criteria.

**Conclusion:**

Most universal adhesives show acceptable clinical performance. There is no significant effect of the adhesive strategy of universal adhesives on their clinical performance according to the results of our meta-analysis.

## Introduction

The advent of adhesive dentistry has revolutionized the field of restorative dentistry. Scientific evidence shows that bonded restorations demonstrate an array of advantages including minimal invasion, better aesthetics, stronger bond strength, reduced microleakage, better distribution of functional stresses and more favourable failures [1, 2].

Kramer and McLean in 1952 [3], were among the first to use glycerophosphoric acid dimethacrylate (GPDM) to bond to dentin. They demonstrated certain alterations in the staining of dentin during histologic examination, that is presently known as the hybrid layer [4]. This pioneering work followed by Buonocore’s discovery of the acid-etch technique has led to major changes in bonding to tooth structure [5]. Over the years, dental adhesives have progressed from three-step systems, to two-step systems and finally to single step systems [6]. The technique or adhesive strategy has also developed from a more time-consuming and technique sensitive total etch system to a simpler self-etch system. The consensus of total-etch versus self-etch strategy for bonding has been widely debated over the past two decades [7–9].

The major disadvantage of the total etch system is that there is a risk of collagen fibril collapse during drying of the demineralized dentin, as a result there is a decrease in bond strength [10–12]. In self-etch adhesive systems, the demineralization of the dental substrates is produced by non-rinsing acidic primers mixed with hydrophilic monomers such as HEMA. These primers dissolve the mineral component of the smear layer and alter the underlying superficial dentin to receive the adhesive into the smear plugs [13]. The low bond strength and the inferior marginal adaptation to the enamel are the drawbacks of self-etch adhesives when compared to total-etch systems [14, 15].

More recently, the introduction of Universal adhesives, also called multimode adhesives can be used with or without acid etching in both enamel and dentin [16–19]. Different researchers have shown that universal adhesives have high bond strength values, and this is due to the acidic monomers that promote chemical bonding to the tooth [18, 20].

Non-carious cervical lesions (NCCLs) are hard tissue defects in the cervical region of the teeth that are not caused by a bacterial agent [21]. Restorations of NCCLs are common in the clinical setting, and represent one of the least durable restorations, with a high rate of retention loss, marginal discoloration and lack of marginal adaptation. The major challenges in restoring NCCLs include the difficulty in controlling moisture and obtaining access to the subgingival margins, as well as selecting the best adhesive strategy [22].

The lack of enamel or macro-mechanical undercuts, high degree of sclerosis and substantial differences in bonding surface makes the hybrid layer formation more difficult on this surface [23, 24]. Phosphoric acid conditioning before self-etch primers has been suggested to improve the retention of these restorations [24]. Since, this effect has not been assessed sufficiently with universal adhesives, the aim of this systematic review was to determine the effects of adhesive strategy (total etch or self-etch) of universal adhesives in non-carious cervical lesions. The Population (P) was patients requiring restoration of non-carious cervical lesions; Intervention (I) was the use of Universal adhesive in Self-etch adhesive strategy; Comparison (C) was the use of Universal adhesive in total etch adhesive strategy and the Outcome (O) was clinical evaluation in terms of Marginal adaptation, Marginal discolouration, Retention, Secondary caries and Post-operative sensitivity.

## Materials and methods

This systematic review was performed following the Preferred Reporting Items for Systematic Reviews (PRISMA 2020) guidelines (http://www.prisma-statement.org) and the Cochrane Handbook of Systematic Reviews of Interventions (Version 6.2.0) [25].

### Protocol registration

The protocol for this systematic review has been registered with the PROSPERO International prospective register of systematic reviews, registry No. CRD42021250429.

### Selection criteria

Only Randomized Controlled Clinical Trials (RCTs) in which non-carious cervical lesions were restored using Universal adhesives in total etch or self etch strategy were included in the systematic review. In case of studies having publications with multiple follow ups, the publication with the longest follow up only was included. In-vitro studies, or studies on cavity designs other than NCCLs were excluded from this systematic review. Studies using adhesives other than universal adhesives, or those not assessing the clinical performance in terms of marginal adaptation, marginal discoloration, retention, secondary caries or post-operative sensitivity were also excluded.

### Search strategy

A detailed search was done in the following databases: Medline (PubMed), Cochrane, Scopus, Web of Science and OpenGrey and clinical trial registry for the identification of relevant studies. The search queries in the database were formulated with the basis of PICO questions in combination with various Boolean operators such as AND, OR. MeSH terms used for the search included “Dentin bonding agent”, “Dental Adhesive”, “acid etching”, “tooth abrasion”, ‘tooth erosion’, “United States Public Health Service”. No language restriction was applied. Related articles and the reference lists of the articles that were retrieved by the search engines were manually checked for possible eligibility (Table 1).

**Table 1:** Search strategy.

Search Engine	Search strategy
PubMed (27 results)	(((((((((((non carious cervical lesions) OR (non-carious cervical lesions)) OR (cervical abrasion)) OR (tooth abrasion)) OR (tooth erosion)) OR (cervical lesions)) OR (NCCL)) OR (NCCLs)) OR (Class V)) OR (Class 5)) AND (((((((((((Dentin Bonding Agent) OR (Dental adhesives) OR (Universal adhesive) OR (Universal bonding agent)) OR (Multimode adhesives)) OR (Multi mode adhesives)) OR (eighth generation bonding agent)) OR (8th generation bonding agent)) OR (8th-generation bonding agent)) OR (eighth-generation bonding agent)) OR (eighth generation adhesive)) OR (8th generation adhesive))))) AND ((((((((((((Self etch) OR (self-etch)) OR (One step self etch)) OR (One-step self etch)) OR (one-step self-etch)) OR (1 step self-etch)) OR (1-step self-etch)) OR (1-step self etch)) OR (self-etch technique)) OR (self-etch strategy)) OR (self-etching)) OR (self etching))) AND (((((((((total etch) OR (acid etching) OR (total-etch)) OR (etch and rinse)) OR (etch & rinse)) OR (etch-and-rinse)) OR (etch-&-rinse)) OR (total etch technique)) OR (total-etch technique)) OR (total-etching) AND ((((((((((post operative sensitivity) OR (post-operative sensitivity)) OR (postoperative sensitivity)) OR (retention)) OR (marginal adaptation)) OR (marginal discolouration)) OR (marginal staining)) OR (United States Public Health Service)) OR (FDI criteria)) OR (secondary caries))
Cochrane (48 results)	#1 (non-carious cervical lesions) OR (cervical abrasion) OR (cervical lesion) OR (NCCL) OR (Class V) (Word variations have been searched)
	#2 MeSH descriptor: [Tooth Abrasion] this term only
	#3 MeSH descriptor: [Tooth Erosion] this term only
	#4 (Universal Adhesives) OR (Universal bonding agents) OR
	(Multimode adhesives) OR (Eighth generation bonding agents) OR (8th generation adhesives) (Word variations have been searched)
	#5 MeSH descriptor: [Dentin-Bonding Agents] this term only
	#6 (self etch) OR (one-step self etch) OR (Self-etching) OR (1 step self-etch) (Word variations have been searched)
	#7 (total etch) OR (etch and rinse) OR (etch & rinse) OR (acid etch) OR (acid etching) (Word variations have been searched)
	#8 MeSH descriptor: [Acid Etching, Dental] this term only
	#9 (FDI criteria) OR (marginal adaptation) OR (marginal discolouration) OR (“United States Public Health Service”) OR (marginal staining) (Word variations have been searched)
	#10 MeSH descriptor: [United States Public Health Service] this term only
	#11 (secondary caries) OR (retention) OR (postoperative sensitivity) OR (post-operative sensitivity) OR (post operative sensitivity) (Word variations have been searched)
	#12 #1 OR #2 OR #3
	#13 #4 OR #5
	#14 #7 OR #8
	#15 #9 OR #10 OR #11
	#16 #12 AND #13 AND #6 AND #14 AND #15
Scopus (43 results)	(TITLE-ABS-KEY (“Non-carious cervical lesion” OR “non carious cervical lesion” OR “cervical abrasion” OR “tooth abrasion” OR “tooth erosion” OR nccl OR nccls)) AND (TITLE-ABS-KEY (“Dentin bonding agent” OR “Dental adhesive” OR “Universal Adhesive” OR “Universal bonding agent” OR “Multimode adhesive” OR “Multi mode adhesive” OR “Eighth generation adhesive” OR “8th generation adhesive” OR “Eighth generation bonding agent” OR “8th generation bonding agent”)) AND (TITLE-ABS-KEY (“self etch” OR {self-etch} OR “self etching” OR {self-etching})) AND (TITLE-ABS-KEY (“total etch” OR {total-etc} OR “total etching” OR {total-etching} OR “acid etch” OR “acid etching” OR “etch and rinse” OR {etch-and-rinse} OR “etching and rinsing” OR {etch-n-rinse})) AND (TITLE-ABS-KEY (“postoperative sensitivity” OR “post operative sensitivity” OR {post-operative sensitivity} OR retention OR “marginal adaptation” OR “marginal discolouration” OR “marginal staining” OR “secondary caries” OR “United States Public Health Service criteria” OR {USPHS criteria} OR {FDI criteria} OR “clinical evaluation” OR “clinical performance”))
Web of Science (25 results)	# 6
	#5 AND #4 AND #3 AND #2 AND #1
	Indexes=SCI-EXPANDED, CPCI-S, ESCI Timespan=All years
	# 5
	(ALL = (postoperative sensitivity OR post-operative sensitivity OR post operative sensitivity OR Retention OR secondary caries OR marginal discolouration OR marginal staining OR marginal adaptation OR United States Public Health Service OR FDI OR clinical evaluation OR clinical performance)) AND LANGUAGE: (English) AND DOCUMENT TYPES: (Article)
	Indexes=SCI-EXPANDED, CPCI-S, ESCI Timespan=All years
	# 4
	(ALL = (self etch OR Self-etch OR self etching)) AND LANGUAGE: (English) AND DOCUMENT TYPES: (Article)
	Indexes=SCI-EXPANDED, CPCI-S, ESCI Timespan=All years
	# 3
	(ALL = (Total etch OR total-etch OR etch and rinse OR acid etching OR total etching OR etching and rinsing)) AND LANGUAGE: (English) AND DOCUMENT TYPES: (Article)
	Indexes=SCI-EXPANDED, CPCI-S, ESCI Timespan=All years
	# 2
	(ALL = (Universal adhesive OR universal bonding agent OR Multimode adhesive OR Multi-mode adhesive OR Eighth generation bonding agent OR 8th generation bonding agent)) AND LANGUAGE: (English) AND DOCUMENT TYPES: (Article)
	Indexes=SCI-EXPANDED, CPCI-S, ESCI Timespan=All years
	# 1
	(All = (Non carious cervical lesion OR Cervical abrasion OR NCCL OR tooth abrasion OR tooth erosion)) AND LANGUAGE: (English) AND DOCUMENT TYPES: (Article)
	Indexes=SCI-EXPANDED, CPCI-S, ESCI Timespan=All years
Open Grey (0 results)	Universal Adhesives AND Non-Carious Cervical Lesions

### Screening process

The search and screening process was carried out by two authors (Krisha Doshi, Pradeep Solete), independently. As a first step, titles and abstracts were analyzed, followed by full text analysis. An in-depth analysis of the articles for inclusion or exclusion was performed by the two authors separately to include in the systematic review. Any disagreement in selecting the articles between the authors was resolved by discussion with the third author (Nivedhitha M. S.), who is a senior professor.

### Data extraction

The studies that fulfilled the inclusion criteria were processed for the extraction of data. The data were recorded as follows: first author and year of publication, type of study, sample size, mean age and gender of the participants, followup period, study groups, number of teeth per group, isolation method, criteria used for clinical evaluation and clinical evaluation scores per group. The information regarding assessment of the risk of bias was also collected. The extraction of the information was done by two independent authors (Krisha Doshi, Pradeep Solete) using a standard form. Incase of any disagreement between the reviewers it was resolved by the third author (Nivedhitha M. S.), who is a senior professor.

### Methodological quality assessment of included studies

The evaluation of the methodological quality of the included studies was assessed using the bias risk assessment tool (RoB 2), described in the Cochrane Handbook of Systematic Reviews of Interventions (Version 6.2.0) [25]. Briefly, five domains were evaluated: (a) bias arising from the randomization process; (b) bias due to deviations from intended interventions; (c) bias due to missing outcome data; (d) bias in measurement of the outcome; and (e) bias in selection of the reported result.

### Meta-analysis

The studies that had 18, 24 or 36 month follow up were included in the meta analysis. Comparison was made separately for each time interval. Meta analyses for FDI and USPHS criteria were conducted separately. Events of retention, marginal adaptation, absence of marginal discoloration, absence of secondary caries and absence of post-operative sensitivity were analysed as dichotomized values. Since both FDI and USPHS criteria were assessed for the above mentioned five factors, a subgroup meta-analysis was conducted.

Estimates of intervention effects were expressed as Risk Ratios (RR) with 95% confidence intervals. The Mantel-Haenszel method which estimates risk ratio after adjusting for confounders was used. Heterogeneity was assessed using I^2^ statistics. Values <25% were considered as low heterogeneity, between 25% and 70% were considered as moderate heterogeneity, >70% were considered as high heterogeneity. Fixed effect model was used if the I^2^ value was <40% and a random effect model was used when the I^2^ value was >40%. The significance level was set at p ≤ 0.05.

Publication bias for the included studies was examined by constructing a funnel plot. Asymmetry of the studies in the funnel plot may reflect publication bias. Data from the included studies were analyzed using RevMan Web (Cochrane’s online review writing platform, The Cochrane Collaboration, Copenhagen, Denmark).

### Certainty of evidence assessment

Grading of Recommendations Assessment, Development and Evaluation (GRADE) approach (GRADEpro GDT software https://gradepro.org) was used to assess certainty of evidence from the meta-analysis which graded evidence into very low, low, moderate or high quality.

## Results

### Search and selection

**Fig. 1:**
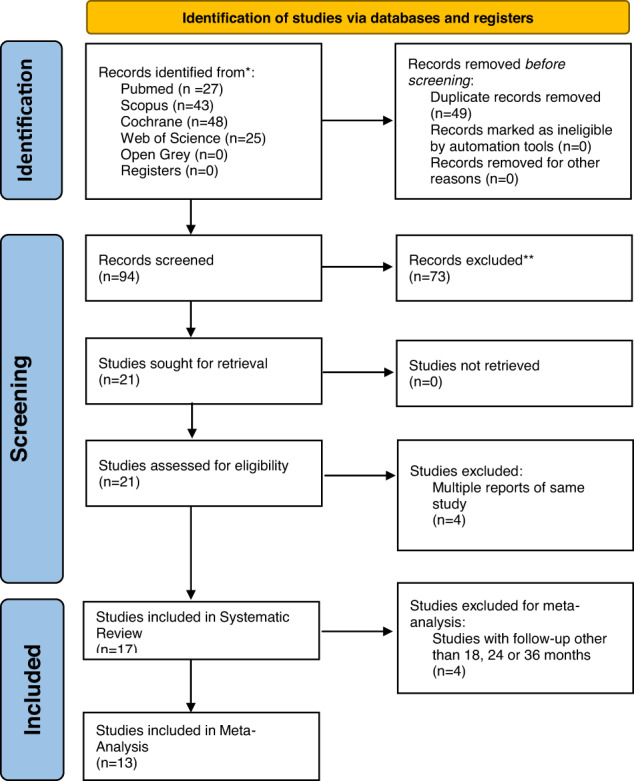
PRISMA Flowchart. Flowchart of Included Studies according to PRISMA guidelines.

Study selection and exclusion is depicted in Fig. 1 and Table 2. Some studies reported multiple reports with different follow-ups. In these cases, the article with the richest information (longest follow-up) was chosen and included in this review. Hence a total of 17 articles were included in the qualitative analysis. 13 of the 17 included articles had a follow up of either 18, 24 or 36 months and were included in the meta-analysis. Sub-group analysis was performed at each of the follow-up periods (18, 24 and 36 months), separately.

**Table 2:** List of excluded studies with reasons.

Author/Year	Reason for exclusion
Mena-serrano et al. (2012) (Mena-Serrano et al. 2013)	Same study as Matos et al. 2020 with different follow up (Matos et al. 2020)
Jorge Perdigao et al. (2014) (Perdigão et al. 2014)	Same study as Matos et al. 2020 with different follow-up (Matos et al. 2020)
Loguercio et al. (2015)(Loguercio et al. 2015)	Same study as Matos et al. 2020 with different follow-up(Matos et al. 2020)
Ruschel et al. (2018)(Ruschel et al. 2018)	Same study as Ruschel et al. 2019 with different follow-up (Ruschel et al. 2019)
Lopes et al. (2016)(Lopes et al. 2016)	6 month follow-up
Haak et al. (2018)(Haak et al. 2018)	6 month follow-up
Manarte-Monteiro et al. (2019)(Manarte-Monteiro et al. 2019)	12 month follow-up
Cruz et al. 2020(Cruz et al. 2020)	6 month follow-up

### Study characteristics

**Fig. 2:**
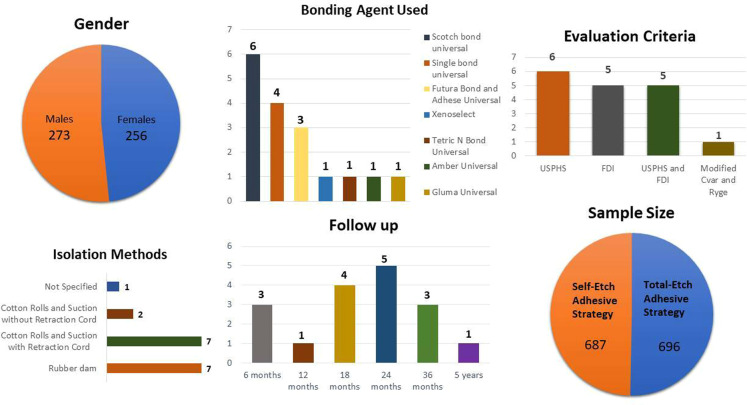
Risk of Bias Graph. Graph representing each risk of bias item presented as percentages across all included studies.

The study characteristics of the included studies are presented in online Supplementary Table 1 and Fig. 2. A total of 2622 teeth were evaluated from 596 patients. 687 restorations were evaluated in the self-etch group while 696 restorations were evaluated in the total-etch group. The age of the patients ranged from 20 to 60 years. 273 of the patients were males wile 256 were females. Scotchbond Universal and Single Bond Universal were the most commonly used adhesives. 17 studies used either USPHS or FDI or both criteria for evaluation of the restorations. An equal number of studies used rubber dam and cotton rolls with gingival retraction cord as the choice of isolation method. The follow-up period ranged from 6 months to 5 years with maximum studies lying in the range of 18–36 months.

Most of the available literature did not purely compare the two adhesive strategies—self-etch and total-etch. They usually included one or more interventions. Some of these interventions included comparison between dry dentin and moist dentin [MD9] within the total-etch group [26–28]; selective enamel etch adhesive strategy [26, 27, 29–31]; effect of dentine roughness [32]; use of optical coherence tomography for evaluation of quality of restorations [33]; addition of copper into the adhesive [34]; comparison with self-etch and total-etch adhesives [30, 31, 35]; effect of diode laser [36]; and catechin-based dentin pretreatment [37]. Only relevant data pertaining to this review was collected from these articles.

### Methodological quality assessment of included studies

**Fig. 3:**
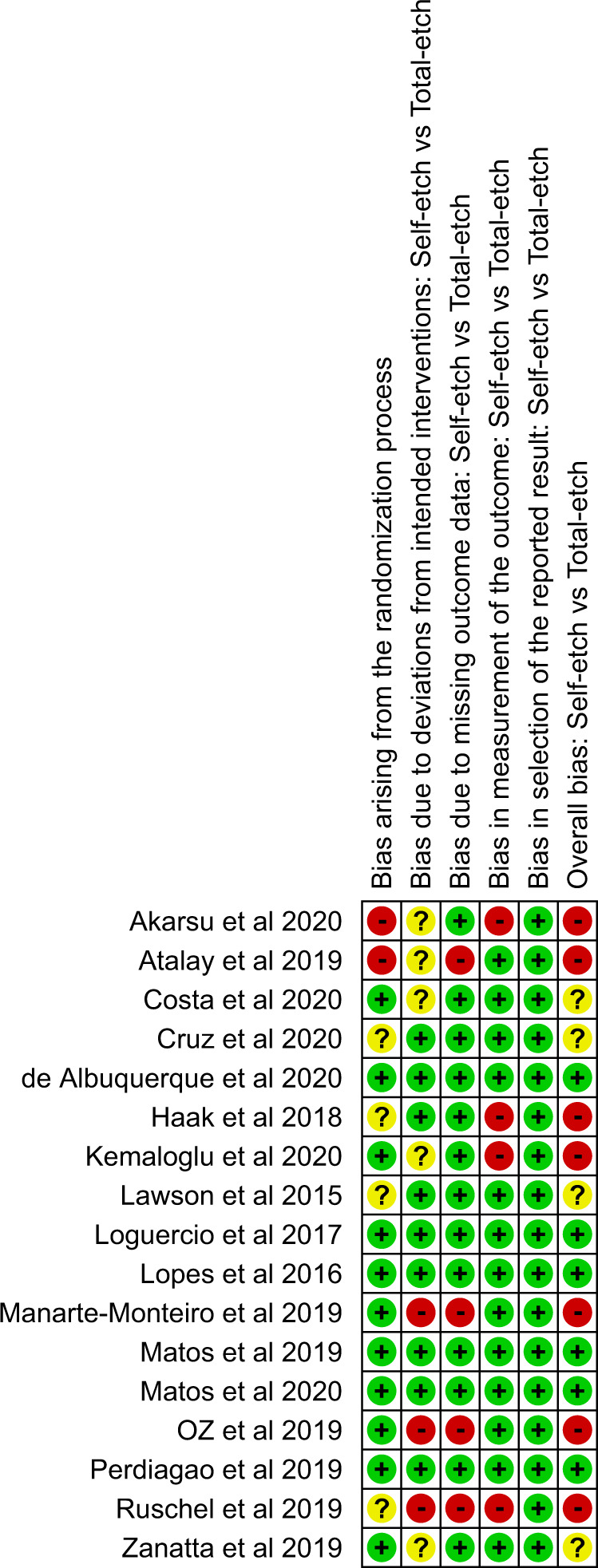
Risk of bias summary. Graph representing the percentage of bias under each category assessed using Cochrane’s risk of bias assessment tool.

**Fig. 4:**
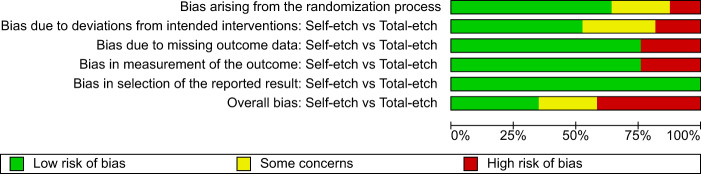
General Characteristics of included studies. Distribution of gender, adhesive, evaluation criteria, Isolation method, follow-up and Sample size among various included studies.

The result of the quality assessment of RCTs of the systematic review is presented as risk of bias summary and risk of bias graph (Figs. 3, 4). As evaluated by the RoB2 tool, 7 [29–31, 33, 36, 38, 39] out of 17 included articles had a high risk of bias, 4 [9, 35, 37, 40] studies showed some concerns and 6 [26–28, 32, 34, 41] studies had a low risk of bias. In specific, the highest risk was seen in the bias due to deviation from the intended interventions domain with 3 studies [30, 31, 39] at high risk and 5 studies [29, 35–38] showing some concerns. In the randomization and allocation concealment domain, 2 studies [29, 36] showed a high risk of bias while 4 studies [9, 33, 39, 40] showed some concerns. 4 studies [29–31, 39] showed a high risk of bias due to missing data and measurement of outcome assessment. All studies showed a low risk of bias in selection of reported results.

### Meta-analyses

A total of thirteen studies which compared self-etch and the total etch adhesive strategy using FDI and USPHS criteria were included in the meta analysis. The forest plot was produced according to the FDI and USPHS criteria for the five outcome measures.

#### Group analysis

**Fig. 5:**
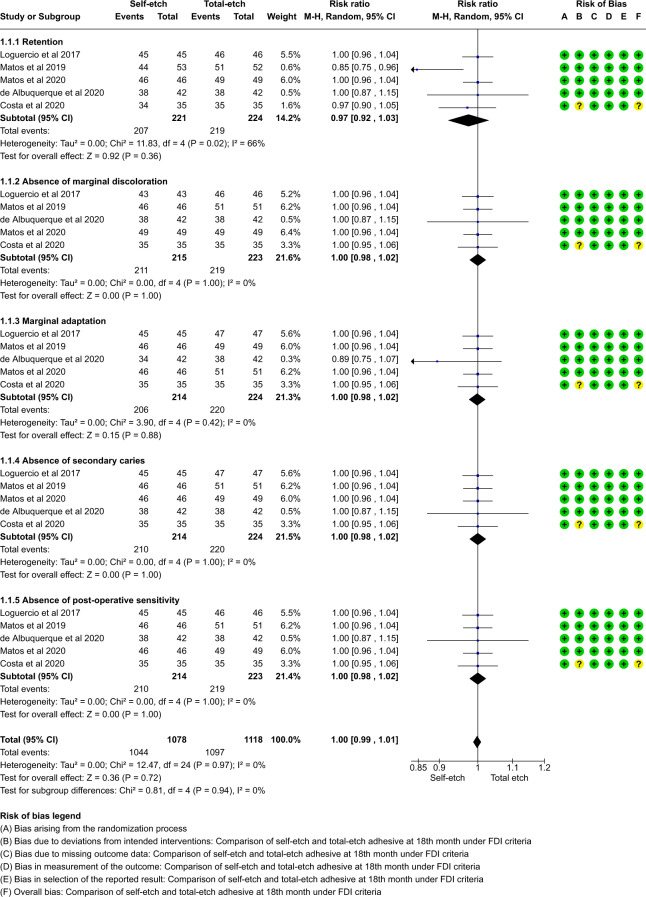
Forest plot representing the pooled events at 18months using FDI criteria. No significant difference observed between total etch and self-etch adhesive strategies for any of the parameters.

**Fig. 6:**
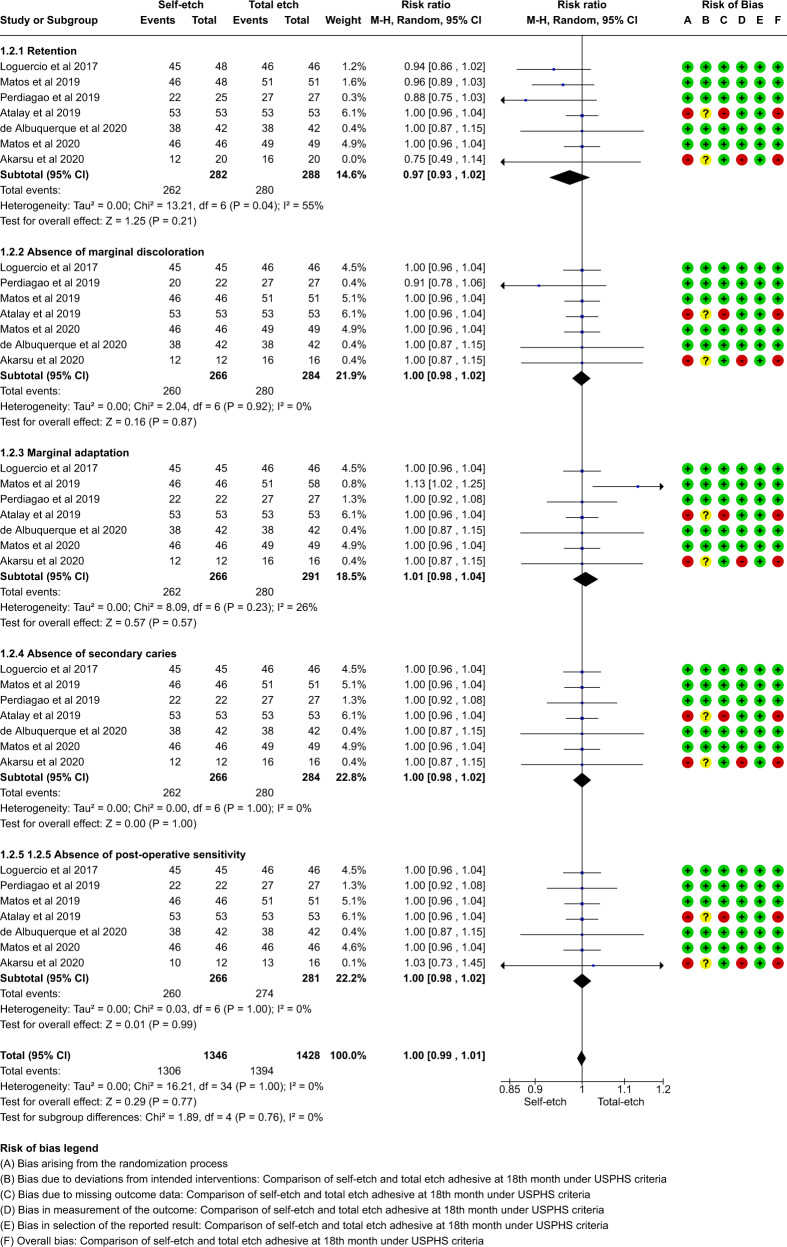
Forest plot representing the pooled events at 18 months using USPHS criteria. No significant difference observed between total etch and self-etch adhesive strategies for any of the parameters.

**Fig. 7:**
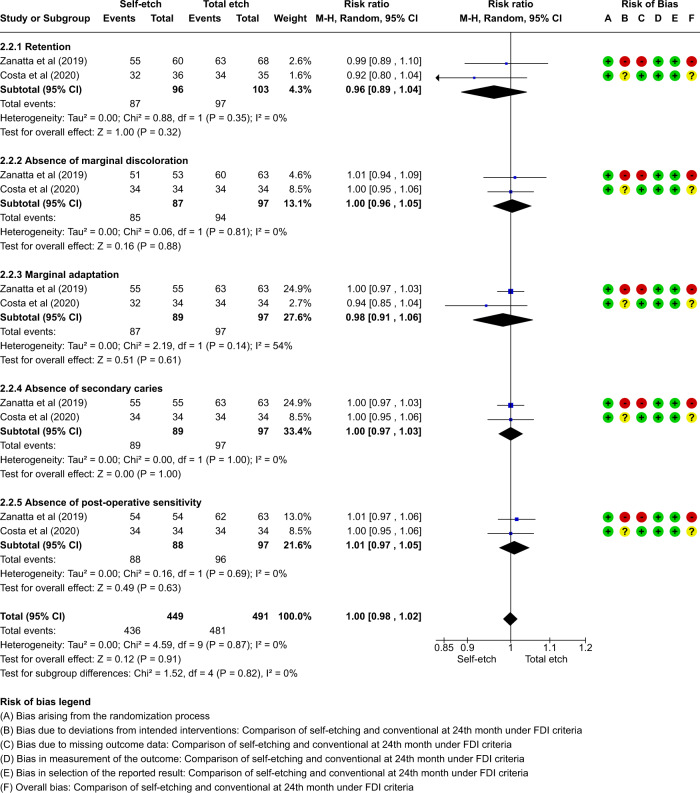
Forest plot representing the pooled events at 24 months using FDI criteria. No significant difference observed between total etch and self-etch adhesive strategies for any of the parameters.

**Fig. 8:**
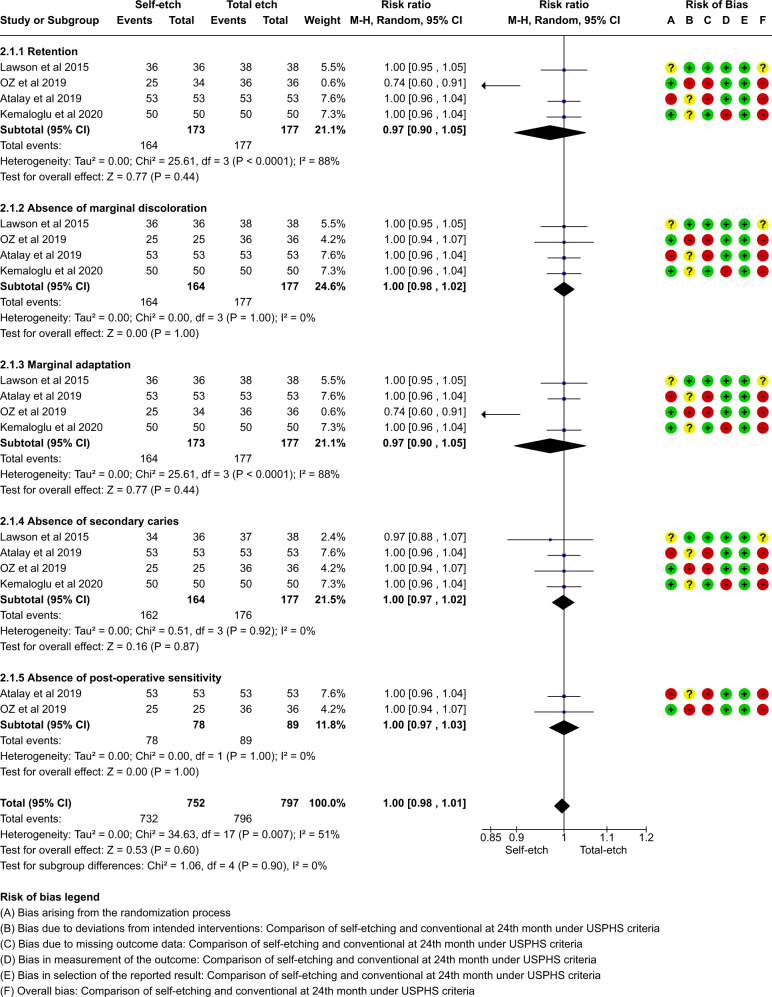
Forest plot representing the pooled events at 24 months using USPHS criteria. No significant difference observed between total etch and self-etch adhesive strategies for any of the parameters.

**Fig. 9:**
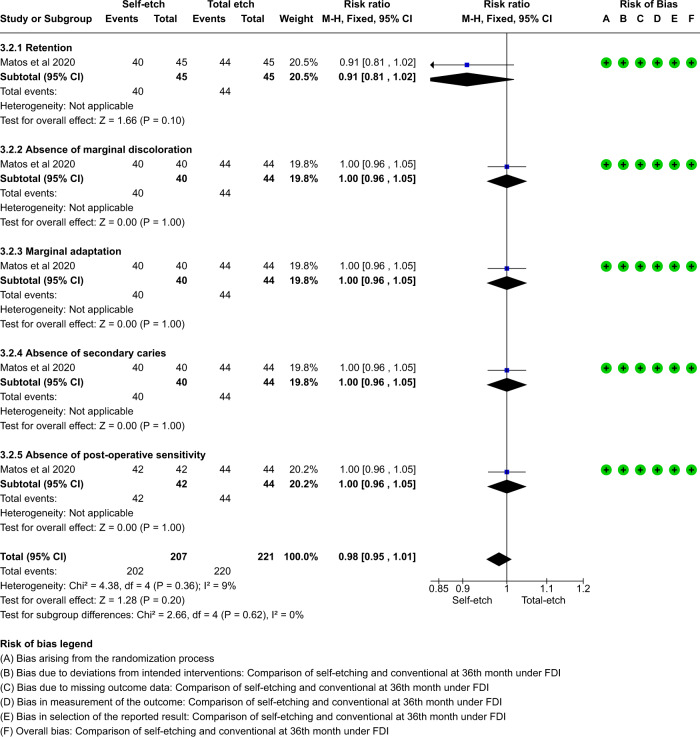
Forest plot representing the pooled events at 36 months using FDI criteria. No significant difference observed between total etch and self-etch adhesive strategies for any of the parameters.

**Fig. 10:**
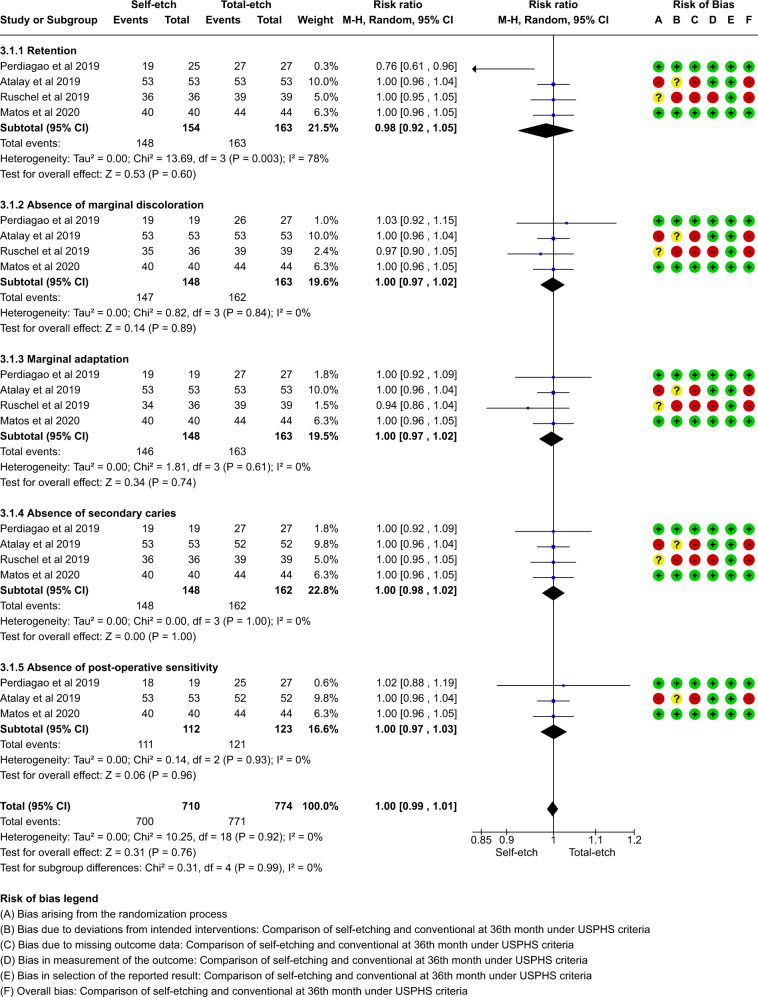
Forest plot representing the pooled events at 36 months using USPHS criteria. No significant difference observed between total etch and self-etch adhesive strategies for any of the parameters.

Overall there was no significant difference between total etch and self- etch adhesive strategies using either the FDI or the USPHS criteria. (p > 0.05, 95% CI, I^2^ value of 0%) at 18, 24 or 36 months. (Figs. 5–10)

#### Subgroup analysis

The outcome of retention, although insignificant, slightly favoured the self-etch strategy at each follow-up for both the FDI and the USPHS criteria.

The outcome of marginal discolouration showed no difference between the two adhesive strategies at any follow-up period for any criteria.

The outcome of marginal adaptation, although insignificant, showed a slight favour towards the self-etch adhesive strategy at the 24 month follow-up for both the criteria. The 36 month follow-up showed no difference between the two adhesive strategies.

The outcome of secondary caries showed no difference between the two adhesive strategies at any follow-up period for any criteria.

The outcome of post-operative sensitivity showed no difference between the two adhesive strategies at any follow-up period for any criteria.

#### Sensitivity analysis

A sensitivity analysis was also done by eliminating the studies with a high risk of bias. No significant difference was observed in the meta-analysis.

### Publication bias

**Fig. 11:**
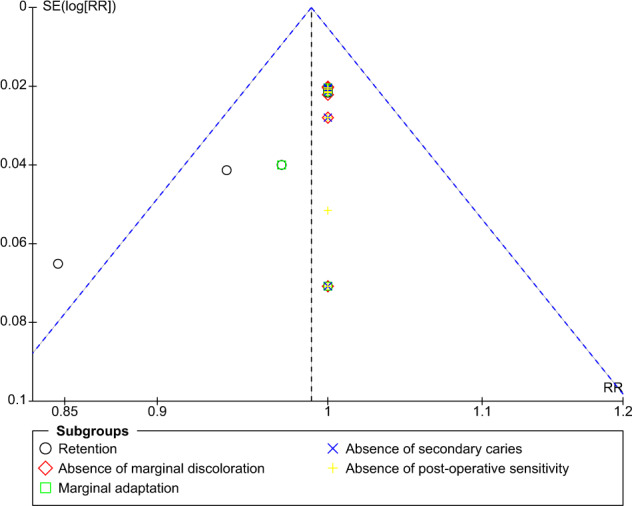
Funnel plot representing asymmetry on analysis of retention across subgroups. It indicates potential publication bias at 18 month follow-up using FDI criteria.

**Fig. 12:**
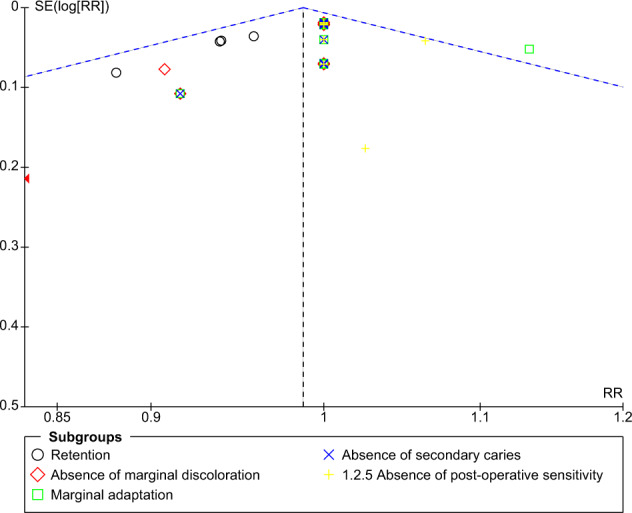
Funnel plot at 18 month follow-up using USPHS criteria. It Represents no asymmetry on analysis across subgroups.

**Fig. 13:**
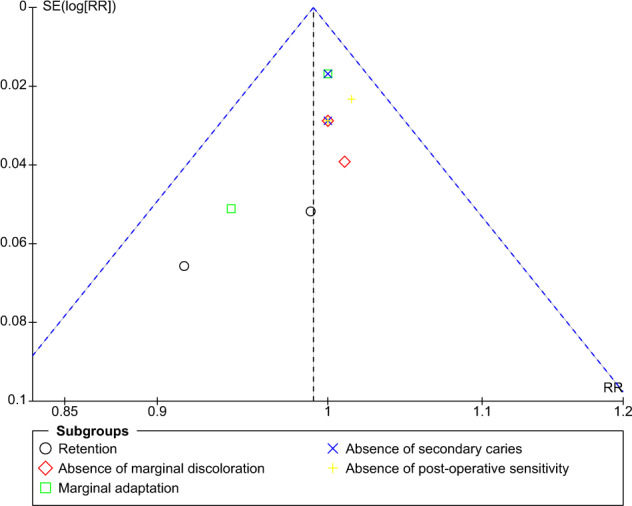
Funnel plot at 24 month follow-up using FDI criteria. It represents no asymmetry on analysis across subgroups.

**Fig. 14:**
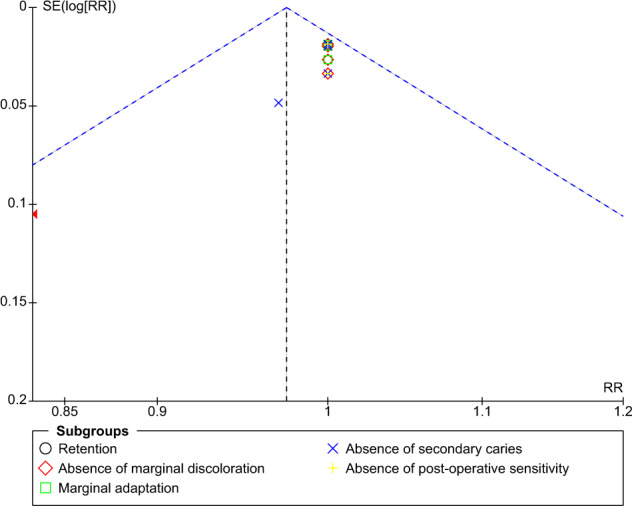
Funnel plot at 24 month follow-up using USPHS criteria. It represents no asymmetry on analysis across subgroups.

**Fig. 15:**
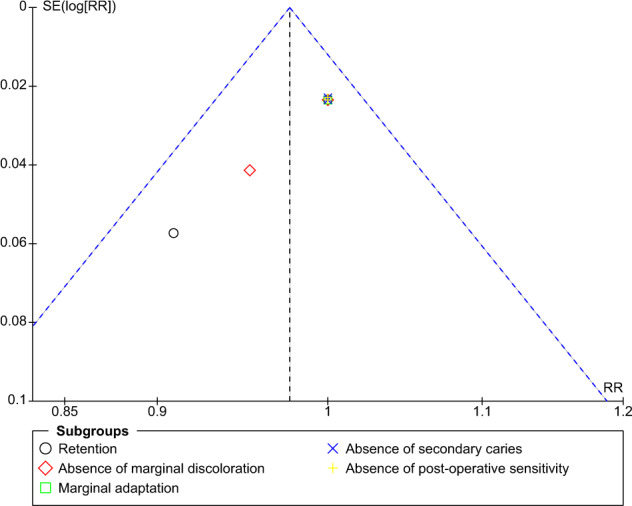
Funnel plot at 36 month follow-up using FDI criteria. It represents no asymmetry on analysis across subgroups.

**Fig. 16:**
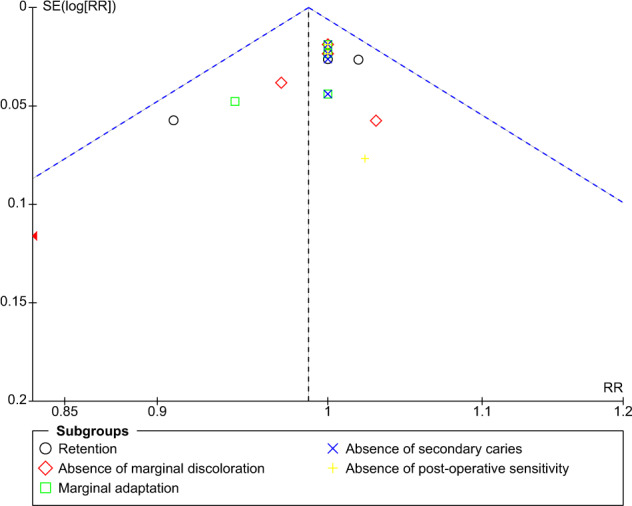
Funnel plot at 36 month follow-up using USPHS criteria. It represents no asymmetry on analysis across subgroups.

The funnel plot for comparison of self-etch and total-etch at 18 month under FDI criteria revealed strongly suspected publication bias in the retention domain (Fig. 11). However, the other domains showed no publication bias (Figs. 12–16).

### Certainty of evidence

The certainty of evidence using GRADEpro revealed different levels of certainty at different follow-up periods using either FDI or USPHS criteria (Refer Tables 3–6).

**Table 3:** Analysis of certainty of evidence.

Follow-up period	Evaluation criteria		No. of studies common to both criteria
	USPHS (No. of studies included)	FDI (No. of studies included)	
18 months	Moderate (7)	High (5)	4
24 month	Low (4)	Moderate (2)	0
36 months	Low (4)	High (1)	1

**Table 4:** GRADEpro assessment of certainty of evidence at 18 month follow-up using FDI and USPHS criteria.

**Table 5:** GRADEpro assessment of certainty of evidence at 24 month follow-up using FDI and USPHS criteria.

**Table 6:** GRADEpro assessment of certainty of evidence at 36 month follow-up using USPHS and FDI criteria.

## Discussion

Clinicians have always been in a dilemma regarding the adhesive strategy to be used while restoring non-carious cervical lesions. Hence, by conducting this systematic review and meta-analysis we synthesized data from available literature on this topic to come to a conclusion as to which adhesive strategy of universal adhesives should be employed in order to optimize clinical performances of composite restorations placed in non-carious cervical lesions. Since RCTs hold a high place in the level of evidence pyramid, all of the 17 studies that were included, were randomized controlled clinical trials.

The two most commonly used criteria for clinical evaluation are United States Public Health Service Criteria (USPHS) and the Federation Dentaire Internationale (FDI) Criteria [42, 43]. The FDI criteria is considered more sensitive as compared to the USPHS criteria [42]. Since combining the two criteria for the meta-analysis may introduce bias, apart from the follow-up durations, we also decided to categorize the studies based on the criteria used for evaluation.

The results of our paper showed good clinical performance of self-etch as well as total etch strategies of universal adhesives in non-carious cervical lesions. There was no statistically significant difference between the two adhesive strategies in terms of retention, marginal adaptation, marginal discolouration, secondary caries or post-operative sensitivity at the 18th, 24th and 36th month follow-ups. However, the retention was slightly higher for the self-etch group at these follow-ups. This may be due to the fact that the total etch strategy is quite technique sensitive. Phosphoric acid etching could nowadays be considered too aggressive for dentin, given all the consequences related to exposure of the vulnerable collagen [44]. If the collagen collapses after etching and rinsing, the resin monomers are unable to infiltrate and form resin tags and hence there is a failure of the micromechanical bonding and retention of the restoration [45]. Another reason could be the role of the 10-MDP molecule which is most commonly present in universal adhesives. This molecule adheres to calcium with mild decalcification of enamel and dentin hydroxyapatite that leads to calcium release and subsequent formation of stable self-assembled MDP-Ca salts in the form of nano layering [46–48]. Hence, when used in self-etch strategy it is capable of providing a simultaneous chemical and micro-mechanical adhesion, leading to improved retention.

Our results are contradictory to those obtained in a previous systematic review and meta-analysis that concluded that retention and risk of post-operative sensitivity were both significantly higher for the total-etch group [49]. This may be because unlike our paper, their meta-analysis combined the results for any criteria used for the clinical evaluation, which may have introduced some kind of bias. Another systematic review [50] that compared the various adhesive strategies of universal adhesives concluded that there was no significant difference between the total-etch and self etch strategy, although it slightly favoured the total-etch strategy in terms of retention, absence of fracture and secondary caries.

Although our results obtained no significant difference between the two adhesive strategies employed, it is worthwhile to know that the certainty of evidence fell from high certainty (in the 18th month followup under the FDI criteria) to moderate-low certainty in the 24 and 36 month follow-ups. There was a difference seen in the certainty of evidence among the two criteria as well. This may be because the number of studies varied at each follow-up as well as in each evaluation criteria. Different studies were evaluated under each criteria at every follow-up period. The certainty of evidence was high for the 36 month followup under the FDI criteria, but only one study was included in this meta-analysis. Hence results of the longer follow-ups must be interpreted with caution. The sensitivity analysis done after eliminating the high risk studies concluded that no significant difference was obtained, and hence the results of the meta-analysis could be considered in spite of including studies with high risk of bias (Tables 3–6).

The funnel plot analysis revealed that there was a strong risk of publication bias in the retention domain at the 18th month follow-up under FDI criteria suggesting that the results must be interpreted with caution and cannot be generalised to a larger population.

The major difference between universal adhesives and traditional one-step SE adhesives is the presence of functional phosphate and/or carboxylate monomers in universal adhesives, for example 10-MDP (10-methacryloyloxydecyl dihydrogen phosphate), GPDM (glycerol-phosphate dimethacrylate), PENTA (dipentaerythritol penta acrylate monophosphate), PAC (polyalkenoic acid copolymer), etc. The high-quality bonding performance can be explained by the presence of the functional monomer 10-MDP in most Universal adhesives. 10-MDP includes a methacrylate polymerizable end, a long hydrophobic 10-carbon chain and a short hydrophilic phos[hate component that is capable of ionizing and interacting with hydroxyapatite. Apart from the ability of nano layering, the presence of the long carbon chain in this molecule has also been reported to contribute to the excellent bonding ability of universal adhesives [51]. The longer chains make the monomer more hydrophobic, which may enhance the chemical interaction with calcium and reduce their degradation [52].

The major strength of our systematic review is that we have evaluated the clinical performance of Universal adhesives in NCCLs separately at various follow-up periods. The follow-up period has an impact on the clinical performance of restorations and hence an appropriate analysis must evaluate data from various studies at the same follow-up period. Another strength is that our meta-analysis evaluates data using different evaluation criteria (USPHS and FDI), separately, since both these criteria have different specifications. Additionally, we used the most recently introduced and highly specific Cochrane’s RoB2 tool for quality assessment, which was introduced in 2019. The previous version had some issues with assessment of bias due to incomplete outcome data and selective reporting of outcomes causing particular difficulties, and confusion over whether studies that were not blinded should automatically be considered to be at high risk of bias. RoB2 takes into account the risk of bias in the meta-analyses and review conclusions as well. Hence, it is a more rigorous assessment of the quality of the clinical trial.

A major limitation of our review is the low and moderate certainty of evidence observed at these short and medium-term follow-ups, the literature suggests that it may take more than 5 years to observe a significant number of events between the treatment groups in clinical settings [53]. Unfortunately, we could not run a meta-analysis for long-term follow-ups since only one study [27] evaluated the NCCL restorations after 5 years of clinical function, and found superior clinical performance for total etch and selective etch compared to self etch strategy.

Hence future prospects must aim at evaluating the clinical performance at longer follow-up periods. This review throws light on the fact that researchers must keep in mind the latest guidelines for evaluation of risk of bias while conducting clinical trials on this subject. This will help in making higher quality research available in the future. Researchers must also focus on maintaining longer follow-ups which would help us evaluate the clinical performance of Universal adhesives in NCCLs in a better way.

## Conclusion

In conclusion, most universal adhesives show acceptable clinical performance. There is no significant effect of the adhesive strategy of universal adhesives on their clinical performance at 18, 24 and 36 month follow-ups according to the results of our meta-analysis. Yet, long term clinical trials are necessary to assess their effect in order to formulate a conclusive analysis.

## Supplementary information

SI Table 1
