# Supplementary material for: Effect of adhesive strategy of universal adhesives in noncarious cervical lesions – an updated systematic review and meta-analysis
Source: BDJ Open. 2023 Feb 13;9:6. doi: 10.1038/s41405-022-00124-6 (PMC9925793; doi:10.1038/s41405-022-00124-6)
Supplement: Supplementary file 1 — SI Table 1 [file 41405_2022_124_MOESM1_ESM.docx]

**Supplementary Table 1:** Representing characteristics of included studies.

| **S.No** | **Author/Yr** | **Type of Study** | **Sample size (pts/ teeth)** | **Age wise Distribution** | **Follow up period** | **Isolation method used** | **Groups (n)** | **Criteria** | **MA** | **MD** | **SC** | **R** | **POS** |
| --- | --- | --- | --- | --- | --- | --- | --- | --- | --- | --- | --- | --- | --- |
| 1. | Lawson et al, 2015 | Double blinded RCT | 37/126 | 60.1 years | 6, 12, 24* months | Rubber dam | Group 2: Scotchbond Universal total-etch | Modified Cvar and Ryge Criteria A (Perfect), B (Fair), C (Unacceptable) | 26- A  12- B | 33- A  5- B | 37- A  1- C | 38/38 | 2.7/10 |
|  |  |  |  |  |  |  | Group 3: Scotchbond Universal self-etch |  | 17- A  19- B | 26- A  10- B | 34- A  2- C | 36/38 | 2.5/10 |
| 2. | L.S. Lopes et al, 2016 | Double blinded RCT | 31/124 | 20-29yrs - 0  30-39yrs - 1  39-49yrs - 6  >49yrs – 24 | 6 months | Rubber Dam | Etch-and-rinse/dentin dry group (ER-D) (XenoSelect Adhesive) | USPHS | 30- A  1- C | 31- A | 31- A | 31- A | 29- A  2- B |
|  |  |  |  |  |  |  |  | FDI | 17- VG  13- GO  1- UN | 31- VG | 31- VG | 30- VG  1- UN | 29- VG  2- GO |
|  |  |  |  |  |  |  | Etch-and-rinse/dentin moist group (ER-M) (XenoSelect Adhesive) | USPHS | 28- A | 27- A  1- B | 28- A | 28- A  3- C | 28- A |
|  |  |  |  |  |  |  |  | FDI | 18- VG  10- GO | 27- VG  1- GO | 28- VG | 28- A  3- PO | 28- VG |
|  |  |  |  |  |  |  | Self-etch group (SET) (XenoSelect Adhesive) | USPHS | 25- A | 23- A 2- B | 25- A | 25- A  6- C | 25- A |
|  |  |  |  |  |  |  |  | FDI | 12- VG  13- GO | 23- VG  2- GO | 2- VG | 25- A  6- PO | 25- VG |
| 3. | Alessandro D. Loguercio et al, 2017 | Double blind RCT | 48/196 | 20-29 yrs :05  30-39 yrs: 08  39-49 yrs: 19  > 49 yrs: 16 | 6, 18* months | Rubber dam | SE (Self etch) (Tertic N Bond Universal Adhesive) | USPHS | 43- A  2- B | 43- A  2- B | 45- A | 45- A  3- C | 45- A |
|  |  |  |  |  |  |  |  | FDI | 21- VG  22- GO  2- SS  3- PO | 33- VG  8- GO  2- SS | 45- VG | 42- VG  3- SS | 45- VG |
|  |  |  |  |  |  |  | ER (Etch-and-Rinse) (Tertic N Bond Universal Adhesive) | USPHS | 44- A  2- B | 45- A  1- B | 46- A | 46- A  2- C | 46- A |
|  |  |  |  |  |  |  |  | FDI | 27- VG  18- GO  2- PO | 43- VG  2- GO  1- SS | 46- VG | 45- VG  1- SS | 46- VG |
| 4. | Rainer Haak et al, 2018 | RCT | 22/88 | Not mentioned | 6 months | Retraction cord | ScotchBond Universal / Self Etch (SU/SE) | FDI | Total Esthetic Score | Total Functional Score | Total Biological score | Cumulative Score | As a part of biological score |
|  |  |  |  |  |  |  |  |  | Acceptable - 100% Unacceptable - 0% | Acceptable - 100% Unacceptable - 0% | Acceptable - 100% Unacceptable - 0% | Acceptable - 100% Unacceptable - 0% |  |
|  |  |  |  |  |  |  | ScotchBond Universal / Etch and Rinse (SU/ER) |  | Total Esthetic Score | Total Functional Score | Total Biological score | Cumulative Score |  |
|  |  |  |  |  |  |  |  |  | Acceptable - 100% Unacceptable - 0% | Acceptable - 100% Unacceptable - 0% | Acceptable - 100% Unacceptable - 0% | Acceptable - 100% Unacceptable - 0% |  |
| 5. | Cansu Atalay et al 2019 | Clinical Trial | 35/165 | 20–29 yrs :6  30–39 yrs: 6  40–49 yrs: 51  50–59 yrs: 48  > 60 yrs: 54 | 6, 12, 18, 24, 36* months | Cotton rolls and suction | SingleBond Universal(SBU), etch-and-rinse mode | Modified USPHS | 47- A  5- B | 45- A  7- B | 52- A | 52- A  1-C | 52- A |
|  |  |  |  |  |  |  | SingleBond Universal(SBU), self-etch mode |  | 36- A  17- B | 36- A  17- B | 53- A | 53- A | 53- A |
| 6. | Thalita P. Matos et al, 2019 | Double blind, RCT | 36/216 | 49 +/- 9 years | 6, 12, 18* months | Rubber dam | Amber Universal Adhesive ERct (Etch-and-rinse without copper nanoparticles) | USPHS | 50- A  1- B | 51- A | 51- A | 51- A  2- C | 51- A |
|  |  |  |  |  |  |  |  | FDI | 47- VG  3- GO  1- SS | 49- VG  2- GO | 51- VG | 51- VG  2- PO | 51- VG |
|  |  |  |  |  |  |  | Amber Universal Adhesive SEct (Self-etch without copper nanoparticles) | USPHS | 43- A  3- B | 46- A | 46- A | 46- A  7- C | 46- A |
|  |  |  |  |  |  |  |  | FDI | 27- VG  16- GO  3- SS | 43- A  3- B | 46- VG | 43- VG  1- GO  2- UN  2- PO | 46- VG |
| 7. | Jorge Perdigão et al, 2019 | Randomized controlled Clinical Trial | 39/134 | 20–29yrs - 3  30–39yrs - 6  40–49yrs -10  50–59yrs -13  60–69yrs – 7 | 6, 18, 36* months | Cotton rolls and retraction cord | Two step ER adhesion strategy (2-ER) (SU) | Modified USPHS | 27- A | 21- A  5- B  1-C | 27- A | 27- A | 25- A  2- C |
|  |  |  |  |  |  |  | One step SE adhesion strategy (1-SE) (SU+SBM) |  | 25- A | 13- A  9- B  3-C | 25- A | 25- A  4- C | 25- A |
| 8. | R.F. Zanatta et al, 2019 | Double blind RCT | 34/152 | 21-40yrs - 5  41-60yrs -27  >60yrs – 2 | 6, 12, 24* months | Cotton rolls, saliva aspirator, and gingival retraction cord | ScotchBond Universal / Etch and Rinse (SU-ER) | FDI | 25- VG  6- GO | 26- VG  3- GO  2- UN | 31- VG | 31- VG  1- PO | 30- VG 1- GO |
|  |  |  |  |  |  |  | ScotchBond Universal / Self Etch (SU-SE) |  | 14- VG  14- GO | 17- VG  8- GO  1- SS  1- UN | 28- VG | 25- VG  3- GO  1- PO | 24- VG  1- GO  1- UN |
| 9. | Fatma Dilsad Oz et al, 2019 | RCT | 20/155 | 20-29yrs - 0  30-39yrs - 3  40-49yrs - 8  50-59yrs - 7  60-65yrs – 2 | 6, 12, 24* months | Cotton rolls | GLUMA Universal- self-etch,(GSE) | Modified USPHS | 3- A  10- B | 4- A  9- B | 13- A | 13- A  5- C | 13- A |
|  |  |  |  |  |  |  | GLUMA Universal- etch-and-rinse (GER) |  | 11- A  7- B | 11- A  7- B | 18- A | 18- A | 18- A |
|  |  |  |  |  |  |  | All-Bond Universal- self-etch (ASE) |  | 5- A  7- B | 5- A  7- B | 12- A | 12- A  4- C | 12- A |
|  |  |  |  |  |  |  | All-Bond Universal- etch-and-rinse (AER) |  | 11- A  7- B | 9- A  9- B | 18- A | 18- A | 18- A |
| 10. | Patrícia Manarte-Monteiro et al, 2019 | Double Blind RCT | 38/210 | 55.5yrs | 12 months | Cotton rolls and retraction cord | G3-Futurabond U_ER | FDI | 28- VG  4- GO | 30- VG  2- GO | 32- VG | 32- VG  1- PO | 32- VG |
|  |  |  |  |  |  |  | G4-Futurabond U_SE |  | 25- VG  7- GO | 27- VG  4- GO  1- SS | 31- VG  1- GO | 31- VG  1- GO  1- PO | 29- VG 3- GO |
|  |  |  |  |  |  |  | G5-Adhese Universal_ER |  | 31- VG  1- GO | 32- VG  1- GO | 32- VG  1- GO | 31- VG  1- GO  1- UN | 29- VG 4- GO |
|  |  |  |  |  |  |  | G6-Adhese Universal_SE |  | 28- VG  6- GO | 30- VG  4- GO | 33- VG  1- GO | 33- VG  1- GO | 32- VG  2- GO |
| 11. | Ruschel et al, 2019 | RCT | 63/203 | 42.6+/- 12.7 years | 36 month | Retraction cord, cotton rolls, lip retractor, and low speed suction | SU_ER (ScotchBond Universal Etch-and-Rinse) | Modified USPHS | 28-A  11-B | 27-A  12-B | 39-A | 39-A |  |
|  |  |  |  |  |  |  | SU_SE (ScotchBond Universal Self Etch) |  | 29-A  5-B  2-C | 22-A  13-B  1-C | 36-A | 36-A |  |
|  |  |  |  |  |  |  | PBE_ER (Prime & Bond Elect Etch-and-Rinse) |  | 33-A  1-B  1-C | 27-A  8-B | 35-A | 35-A |  |
|  |  |  |  |  |  |  | PBE_SE (Prime & Bond Elect Self Etch |  | 28-A  8-B  1-C | 32-A  5-B | 37-A | 37-A  3-C |  |
| 12. | S. Akarsu et al, 2020 | Clinical Trial | 20/80 | 38-62 yrs | 6, 18* months | Retractor, cotton roll, saliva ejector and retraction cord | G 1 (SBU Self‑etch) | Modified USPHS | 10- A  2- B | 9- A  3- B | 12- A | 12- A  8- C | 8- A  2- B  2- C |
|  |  |  |  |  |  |  | G 2 (SBU Etch‑and‑rinse) |  | 14- A  2- B | 14- A  2- B | 16- A | 16- A  4- C | 5- A  8- B  3- C |
| 13. | CAGA Costa et al, 2020 | Blinded RCT | 33/156 | 22-66yrs | 6, 12, 18, 24* months | Labial retractor, cotton rolls, and retraction cord. | SBU ER -etch-and-rinse; | FDI | 33- VG+GO 1- SS | 34- VG+GO | 34- VG+GO | 33- VG +GO  1- SS  1- PO | 34- VG+GO |
|  |  |  |  |  |  |  | SBU SE, self-etch |  | 33- VG+GO  1- UN | 30- VG+GO  4- SS | 34- VG+GO | 32- VG +GO  2- SS  1- PO | 34- VG+GO |
| 14. | Thalita de Paris Matosa et al, 2020 | Double Blinded RCT | 39/200 | 20-29yrs- 5  30-39yrs -12  40-49yrs -12  >49yrs – 10 | 6, 18, 36months, 5 years* | Rubber Dam | SU-TEm: etch-and-rinse + moist dentin | USPHS | 32- A  8- B | 37- A  2- B  1- C | 40- A | 40- A  3- C | 40- A |
|  |  |  |  |  |  |  |  | FDI | 29- VG  7- GO  4- SS | 35- VG  2- GO  3- SS | 40- VG | 40- VG  3- PO | 40- VG |
|  |  |  |  |  |  |  | SU-TEd: etch-and-rinse + dry dentin | USPHS | 34- A  6- B | 37- A  3- B | 40- A | 40- A  3- C | 40- A |
|  |  |  |  |  |  |  |  | FDI | 30- VG  6- GO  4- SS | 34- VG  3- GO  3- SS | 40- VG | 40- VG  3- PO | 40- VG |
|  |  |  |  |  |  |  | SU-SE: self-etch | USPHS | 25- A  10- B | 29- A  6- B | 35- A | 35- A  8- C | 35- A |
|  |  |  |  |  |  |  |  | FDI | 18- VG  5- GO  12- SS | 25- VG  10- SS | 35- VG | 35- VG  8- PO | 35- VG |
| 15. | Hande Kemaloglu et al, 2020 | RCT | 25/100 | 45-60yrs | 6, 12, 24* months | Not specified | 1: SBU Adhesive in SE mode +Charisma Opal Flow | USPHS | 23- A  2- B | 19- A  6- B | 25- A | 25- A |  |
|  |  |  |  |  |  |  | 2: SBU Adhesive in ER mode + Charisma Opal Flow |  | 23- A  2- B | 22- A  3- B | 25- A | 25- A |  |
|  |  |  |  |  |  |  | 3: SBU Adhesive in SE mode + Gaenial Universal Flow |  | 24- A  1- B | 23- A  2- B | 25- A | 25- A |  |
|  |  |  |  |  |  |  | 4: SBU Adhesive in ER mode + Gaenial Universal Flow |  | 23- A  2- B | 23- A  2- B | 25- A | 25- A |  |
| 16. | de Albuquerque et al, 2020 | Multi-center RCT | 50/200 | 20-29yrs - 3  30-39yrs -3  39-49yrs -20  >49yrs - 24 | 18  months | Rubber Dam | Self Etch Group | USPHS | 32-A  6-B | 36-A  2-B | 38-A | 38-A  4-C | 38-A |
|  |  |  |  |  |  |  |  | FDI | 25-VG  10-GO  3-SS | 32-VG  6-GO | 38-VG | 38-VG  4-PO | 38-VG |
|  |  |  |  |  |  |  | Etch and Rinse Dry Dentin Group  (ERDry) | USPHS | 38-A  2-B | 40-A | 40-A | 40-A  3-C | 40-A |
|  |  |  |  |  |  |  |  | FDI | 34-VG  6- GO | 39-VG  1-GO | 40-VG | 40-VG  3-PO | 40-VG |
|  |  |  |  |  |  |  | Etch and Rinse Wet Dentin Group  (ERWet) | USPHS | 36-A  2- B | 38-A | 38-A | 38-A  4-C | 38-A |
|  |  |  |  |  |  |  |  | FDI | 32-VG  6- GO | 37-VG  1-GO | 38-VG | 38-VG  4 -PO | 38-VG |
| 17. | Cruz et al, 2020 | RCT | 26/117 | Not mentioned | 6 months | Rubber dam | Etch and Rinse Group | FDI | 39-VG  7-GO  3-SS  1-PO | 48-VG  1-SS  1-PO | 59-VG | 50-VG  9-PO | 35-VG  13-GO  2-SS |
|  |  |  |  |  |  |  | Self-etch Group |  | 57-VG  1-GO | 54-VG  1-SS  1-UN  2-PO | 58-VG | 58-VG | 45-VG  12-GO  1-SS |

‘*’ indicates the follow-up period at which data was evaluated.

USPHS Criteria : A-Alpha, B-Bravo, C-Charlie.

FDI Criteria: VG- Very Good, GO-Good, SS-Sufficient/Satisfactory, UN-Unsatisfactory, PO-Poor.

SU: Scotchbond Universal; SBU: SingleBond Universal; SBM: Scotchbond Multipurpose; EGCG: Epigallocatechin-3-gallate.

ER: Etch-and-Rinse, SE: Self-Etch
